# Association between pre-pregnancy BMI and preterm birth in Chinese women: a retrospective study

**DOI:** 10.1080/07853890.2026.2639163

**Published:** 2026-03-11

**Authors:** Qing Li, Xinyi Lu, Jun Zhang, Tao Duan

**Affiliations:** ^a^ Department of Obstetrics, Shanghai First Maternity and Infant Hospital, Tongji University, Shanghai, China; ^b^ Department of Biostatistics, School of Public Health, Fudan University, Shanghai, China; ^c^ Xinhua Hospital, Shanghai Jiao-Tong University School of Medicine, Shanghai, China; ^d^ Department of Obstetrics, Shanghai First Maternity and Infant Hospital, Shanghai East Hospital, Tongji University, Shanghai, China

**Keywords:** pre-pregnancy BMI, preterm birth, underweight, threshold, Chinese

## Abstract

**Objective:**

To explore the association between pre-pregnancy BMI (PPBMI) and preterm birth (PTB) across the Chinese population.

**Methods:**

A total of 72,827 singleton pregnancies were included in this retrospective cohort study. Women were divided into 4 groups according to PPBMI (kg/m^2^): underweight (<18.5), normal weight (18.5-23.9), overweight (24-27.9) and obesity (≥28); Underweight was further categorized into severe (<16.5), moderate (16.5-17.4) and mild (17.5-18.4) underweight. PTB was grouped as spontaneous and medically indicated PTB based on clinical presentation, or grouped as 24-27, 28-31 and 32-36 weeks based on gestational age. Binary logistic regression was applied to estimate odds ratios for PTB, adjusted for potential confounders. Restricted cubic spline was used to assess potential nonlinear relationships between PPBMI and PTB.

**Results:**

The overall PTB rate was 4.8% (*n* = 3, 478). Compared with the normal weight group, the risks of PTB were similar across underweight categories: aOR = 1.10(95% CI 0.97-1.24) in mild, aOR = 0.96 (95% CI 0.79-1.18) in moderate and aOR = 1.16 (95% CI 0.85-1.60) in the severe underweight group; The risks of PTB were higher both in the overweight (aOR, 1.32; 95% CI, 1.20-1.45) and obesity (aOR, 1.47; 95% CI, 1.24-1.74) group; the trends associated with PPBMI in the risks for medically indicated PTB, spontaneous PTB and PTB at different weeks of gestation were generally consistent with those observed in overall PTB. The association between PPBMI and PTB in the study population exhibited a J-shaped curve, and the nadir of PTB risk corresponded to a PPBMI of approximately 18.5–21.5 kg/m^2^, with a potential risk threshold around 24.5 kg/m^2^.

**Conclusions:**

In Chinese population, pre-pregnancy underweight and severity of underweight were not significantly associated with the risk of PTB; while overweight and obesity increased the risk of PTB. The nadir of PTB risk corresponded to a PPBMI of approximately 18.5–21.5 kg/m^2^, with a potential risk threshold around 24.5 kg/m^2^.

## KEY MESSAGES

In Chinese population, pre-pregnancy underweight and severity of underweight were not significantly associated with the risk of PTB; while both pre-pregnancy overweight and obesity increased the risk of PTB. The association between PPBMI and PTB exhibited a J-shaped curve, clinicians may recommend maintaining a PPBMI of approximately 18.5–21.5 kg/m^2^ and provide comprehensive prenatal screening and education on PTB prevention when PPBMI exceeds 24.5 kg/m^2^.

## Introduction

Preterm birth (PTB) is a leading cause of neonates and children (under 5 years old) mortality [1,2], and is also a predictor of long-term medical and neurodevelopmental sequelae for the neonate[1,3]. Despite the number of babies born preterm each year decreased from 13.8 million in 2010 to 13.4 million in 2020, little change was observed in the global PTB rate in the last decade, from to 9.8% in 2010 to 9.9% in 2020 [4]. PTB remains a major clinical challenge due to its multifactorial etiology and the absence of a definitive cure, imposing a substantial economic and emotional burden[5]. Maternal demographic characteristics associated with PTB include low socioeconomic and advanced maternal age[6,7]. Previous studies have demonstrated that a history of preterm birth is a strong predictor for recurrent preterm delivery[7,8].

Pre-pregnancy BMI (PPBMI), a common indicator of nutritional status, is a well-recognized modifiable risk factor for PTB [9], with both low and high PPBMI have been shown to associate with PTB. The distribution of PPBMI among reproductive women is characterized by distinct profiles in the United States and China. Data from the National Center for Health Statistics (2013-2018) indicate a substantial and rising prevalence of pre-pregnancy obesity (≥30kg/m^2^) in the U.S., increasing from 26.0% to 30.5%, while the prevalence of pre-pregnancy underweight (<18.5 kg/m^2^) remained consistently low among non-Asian American women, ranging from 2.4% to 3.9% [10]. In contrast, a large multicenter retrospective cohort study (*n* = 117,738) conducted across 150 maternity hospitals in China (2015-2018) reported that only 3.0% of women were classified as obese (≥28kg/m^2^), while 13.6% were underweight [11]. Large studies in U.S. and European cohorts have demonstrated a significant association between pre-pregnancy obesity and PTB [12,13]; Meanwhile, pre-pregnancy underweight was observed to be associated with PTB [13,14], and increasing severity of underweight was associated with increasing risk [15,16]. A large cohort study of 536,098 pregnant women in rural China demonstrated a significant association between preconception underweight and PTB, however, neither overweight nor obesity was significantly associated with PTB risk [17]; The study adopted data from the National Free Preconception Checkups Project (NFPCP) in China during 2010–2012, and included women who had successfully conceived within 1 year after the preconception examination. Two cohort studies in China—a prospective study (*n* = 51,125) in Southwest China (2013–2018) [18] and a retrospective study (*n* = 36,596) in Shanghai [19], which applied Asian-specific BMI cutoffs (23.0 for overweight, 27.5 for obesity) [20], found no increased risk of PTB in underweight group among Chinese; Meanwhile, both overweight and obesity were significantly associated with an elevated risk of PTB. Recent studies in Chinese populations have reported inconsistent findings regarding the association between PPBMI and PTB risk. Consequently, a large-scale study employing a multidimensional framework is warranted. This investigation should adopt stratified analyses of underweight severity and PTB subgroups (defined by clinical presentation and gestational age) to generate findings that are directly applicable to clinical practice in China. Previous studies have indicated that low gestational weight gain was associated with increased PTB risk [21]; likewise, gestational complications such as gestational diabetes mellitus and gestational hypertension were established contributors to PTB [7,22,23]. This study designed to investigate the association between pre-pregnancy factors, particularly PPBMI, and PTB risk. The aim is to provide evidence-based PTB risk alert for women coming for preconception counseling or early prenatal care.

## Materials and methods

### Study population

This retrospective study was conducted at Shanghai First Maternity and Infant Hospital (SFMIH), Tongji University School of Medicine. SFMIH is currently the largest obstetric care center in Shanghai, China, with approximately 20,000 deliveries annually. All women who underwent routine prenatal visits and delivered at SFMIH from January 2019 to December 2022 were included in the study. The inclusion criterion was singleton-pregnant women (≥18years) with live-born infants who delivered at ≥24 gestational weeks. All eligible singleton pregnancy episodes were included in the analysis. The exclusion criteria were non-Chinese citizens, women who had fetuses with major anomalies, or who required intrauterine intervention due to fetal-related issues, or missing data on pre-pregnancy weight.

### Data collection and definition

This study utilized data from an investigator-initiated trial entitled ‘Association between pre-pregnancy BMI and adverse pregnancy outcomes’. Ethical approval was obtained from the SFMIH Ethics Committee (initial approval: KS23202, May 24, 2023; renewed approval following protocol amendment: KS25297, June 3, 2025).

The study adhered to the principles of the Declaration of Helsinki. Informed consent was waived by the ethics committee due to the study’s minimal risk nature and its impracticability without such a waiver. All data, including maternal demographics, medical and prenatal history, and postpartum and neonatal details, were extracted from the anonymized electronic medical records of SFMIH. Data extraction and cleaning were conducted from May 2023 to July 2025. The statistical analysis phase, which included both exploratory and formal modeling, commenced in January 2025 and was completed in August 2025.

The primary explanatory variable was PPBMI, calculated as the pre-pregnancy weight divided by the height squared (kg/m^2^), which was typically documented during the initial prenatal visit by self-report. Underweight BMI was defined as <18.5 kg/m^2^ and further categorized into severe (<16.5), moderate (16.5-17.4), and mild (17.5-18.4), based on the 1^st^ percentile (16.5) and the 5^th^ percentile (17.5) of the PPBMI within the study population. We applied the threshold values for overweight (24.0) and obesity (28.0) according to clinical practice guidelines of the Chinese Maternal and Child Health Care System, based on the average BMI for the general Chinese population [24]. The primary outcome of the study was PTB, defined as those that occur at less than 37 gestational weeks. Medically indicated PTB was defined as a delivery initiated by cesarean section or labor induction due to specific maternal or fetal complications. These included, but were not limited to, preeclampsia/eclampsia, fetal growth restriction with abnormal Doppler findings, non-reassuring fetal status, and significant placenta previa; Spontaneous PTB was defined as delivery following preterm labor or preterm premature rupture of membranes [25]. PTB was also further divided into extremely preterm (24-27 weeks), very preterm (28-31 weeks) and moderate to late preterm (32-36 weeks) based on gestational age. Detailed definitions of the relevant confounding factors are provided in Supplement 1, including maternal age, uterine malformation, insurance status, type of conception, parity, ethnicity, pregestational diabetes mellitus (PGDM), and chronic hypertension.

### Statistical analysis

Continuous variables were summarized as mean (standard deviation, SD) or median (IQR), as appropriate, and categorical variables were presented as number (percentage). Bivariate comparisons of continuous variables were performed using analysis of variance (Kruskal-Wallis H), and comparisons of categorical variables using the Chi-squared test. Univariable analyses assessing the associations between pre-pregnancy factors and PTB risk were performed, with odds ratios (ORs) and 95% confidence intervals (CIs) estimated. Binary logistic regression was applied to estimate odds ratios for PTB, adjusting for potential confounders as necessary. PPBMI was modeled by using restricted cubic splines (RCS) [26] with 4 knots to assess potential nonlinear relationships. A PPBMI of 18.5 kg/m^2^ was chosen as the reference in the study group. Pooled ORs across the range of PPBMI were calculated with PPBMI = 18.5 kg/m^2^ set as the reference. PPBMI was grouped into categories of 1 kg/m^2^ each, ranging from 16.5 to 27.5 kg/m^2^ or greater; PPBMI < 16.5 kg/m^2^ was grouped together. The adjusted odds ratios (aORs) for PTB were calculated as per PPBMI category for women in the study population vs. all the other women. Protective PPBMI was defined as all PPBMI categories with a statistically significant protective association (OR < 1) for PTB. If a PPBMI category with a nonsignificant association was between 2 significant estimates with an OR of less than 1, that category was included in the protective PPBMI range.

**Figure 1.**
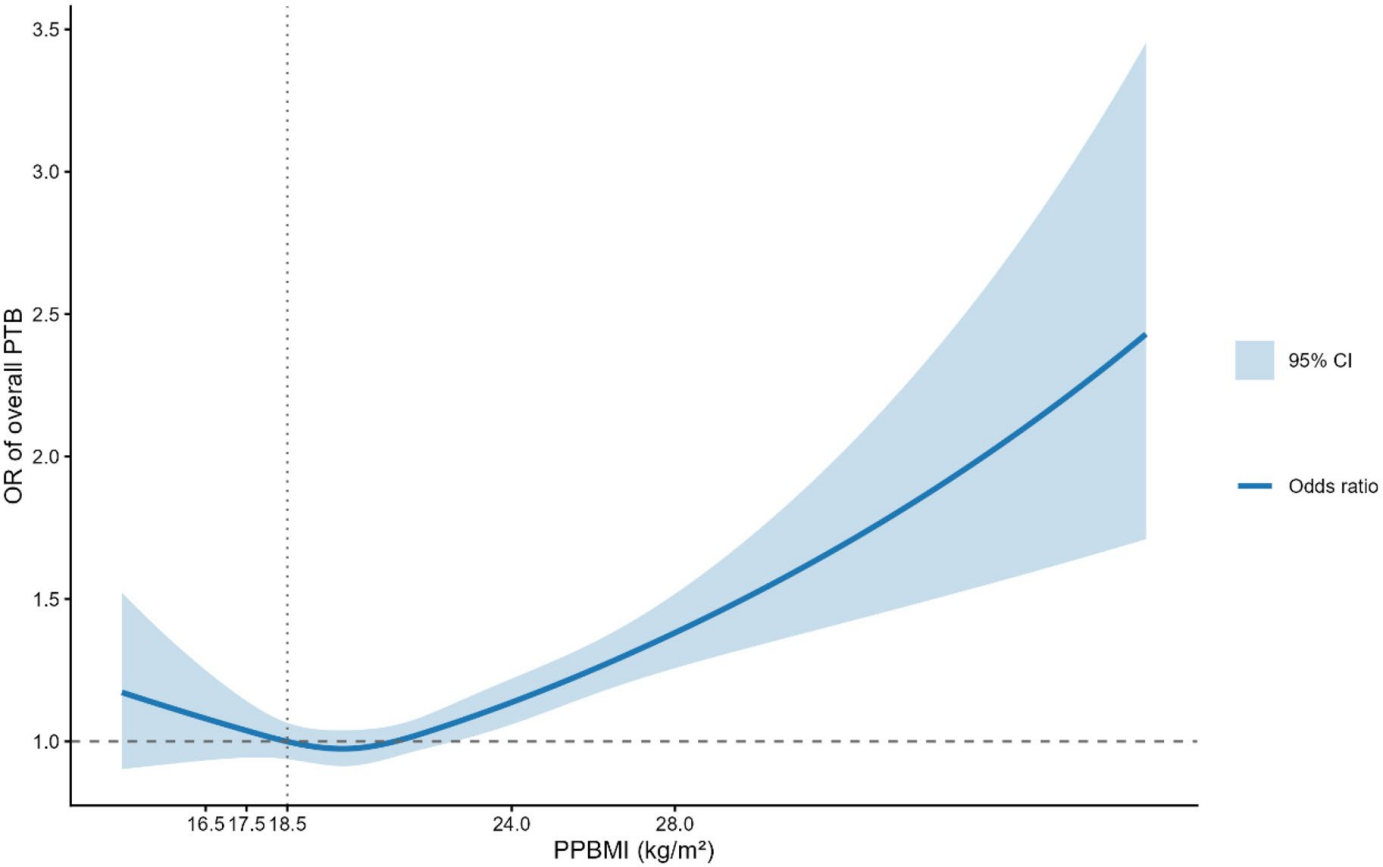
ORs (95% CIs) of overall PTB according to PPBMI in the study population. A PPBMI of 18.5 kg/m^2^ was chosen as the reference in the study group. Analyses were adjusted for maternal age, uterine malformation, insurance status, type of conception, parity, ethnicity, pregestational diabetes mellitus and chronic hypertension. PPBMI: pre-pregnancy BMI; PTB: preterm birth.

All the data were analyzed with SPSS software (version 22; IBM Corp., Chicago, IL), and Figure 1 was generated using R (version 4.3.3) with the rms and ggplot2 packages. A p-value <0.05 or a 95% confidence interval (CI) not including 1 was considered statistically significant.

## Results

After restricting the sample according to the inclusion and exclusion criteria, we obtained data on 72,827 singleton pregnancies for 71,827 unique women. The prevalence rates of underweight, normal weight, overweight and obesity in this study were 13.1%, 70.6%, 13.3%, and 3.0%, respectively. Maternal age varied significantly by PPBMI category (*p* < 0.05), showing a positive trend with higher PPBMI; Women in the underweight group were younger, had a lower proportion of advanced maternal age (≥35 y) and exhibited higher prevalences of nulliparity and natural conception (*p* < 0.05). Higher PPBMI was associated with increased frequencies of PGDM, chronic hypertension, and cesarean delivery (*p* < 0.05). Gestational age at delivery differed significantly across PPBMI groups (*p* < 0.05), with a decreasing trend observed in higher PPBMI categories; Neonatal birthweight differed significantly across PPBMI groups (*p* < 0.05), showing a positive trend with higher PPBMI. Other baseline and obstetric characteristics of the patients included in the study are shown in Table 1. A very small proportion of women (<0.5%) had documented tobacco use or alcohol consumption during pregnancy.

**Table 1.** Baseline and obstetric characteristics of the study population.

	Underweight	Normal weight	Overweight	Obesity	
	(*n* = 9519)	(*n* = 51448)	(*n* = 9671)	(*n* = 2189)	P value
Maternal age a	30.2 (3.6)	31.2 (3.8)	31.9 (4.0)	31.5 (4.1)	<0.001
Maternal age group, n (%)					<0.001
18-29 y	4241 (44.6)	17207 (33.4)	2803 (29.0)	686 (31.3)	
30-34 y	4241 (44.6)	24936 (48.5)	4514 (46.7)	1019 (46.6)	
≥35 y	1037 (10.8)	9305 (18.1)	2354 (24.3)	484 (22.1)	
Han ethnicity, n (%)	9372 (98.5)	50660 (98.5)	9504 (98.3)	2148 (98.1)	0.338
Insurance, n (%)	7935 (83.4)	44517 (86.5)	8186 (84.6)	1773 (81.0)	<0.001
Nulliparous, n (%)	7614 (80.0)	37833 (73.5)	6629 (68.5)	1514 (69.2)	<0.001
Natural conception, n (%)	9016 (94.7)	47242 (91.8)	8490 (87.8)	1860 (85.0)	<0.001
Uterine malformation	60 (0.6)	448 (0.9)	90 (0.9)	24 (1.1)	0.046
PGDM, n (%)	6 (0.1)	51 (0.1)	83 (0.9)	77 (3.5)	<0.001
Chronic hypertension, n (%)	7 (0.1)	99 (0.2)	118 (1.2)	118 (5.4)	<0.001
Cesarean delivery, n (%)	3352 (35.2)	21918 (42.6)	5465 (56.5)	1413 (64.6)	<0.001
Gestational week ^b^	39.3 (38.6, 40.0)	39.3 (38.6, 40.1)	39.1 (38.4, 40.0)	39.0 (38.3, 39.9)	<0.001
Neonatal birthweight ^a^	3187.2 (406.1)	3310.5 (427.8)	3394.0 (487.8)	3430.3 (524.1)	<0.001
Neonatal sex, female, n (%)	4615 (48.5)	24518 (47.7)	4602 (47.6)	1083 (49.5)	0.184

^a^Data are given as the mean (SD). ^b^Data are given as the median (Q1, Q3).

PGDM: pregestational diabetes mellitus.

Underweight: <18.5 kg/m^2^, normal weight: 18.5-23.9 kg/m^2^, overweight: 24-27.9 kg/m^2^, obesity: ≥28 kg/m^2^.

In univariable analyses, multiple factors—including advancing maternal age, uterine malformation, lack of medical insurance, non-natural conception, multiparity, PGDM, and chronic hypertension—were significantly associated with a higher PTB risk. However, ethnic minority status showed no significant association (*p* > 0.05) (Table 1 and Supplementary 2).

The overall PTB rate was 4.8% (*n* = 3, 478), with an increasing trend observed with higher PPBMI: 4.5% (*n* = 431) in underweight, 4.5% (*n* = 2, 291) in normal weight, 6.1% (*n* = 594) in overweight, 7.4% (*n* = 162) in obesity. Among underweight women, no significant trend in the incidence of all PTB was observed with increasing severity of maternal underweight: 4.7% in mild (*n* = 290), 4.1% in moderate (*n* = 100) and 4.9% in severe underweight (*n* = 41). The adjusted ORs were aOR =1.10 (95% CI 0.97-1.24) in mild, aOR = 0.96 (95% CI 0.79-1.18) in moderate and aOR =1.16 (95% CI 0.85-1.60) in the severe underweight group (Table 2). Compared with the normal weight group, the risks of all PTB were higher in the overweight (aOR, 1.32; 95% CI, 1.20-1.45) and obesity (aOR, 1.47; 95% CI, 1.24-1.74) group. 28.8% of preterm deliveries were medically indicated (*n* = 1, 003), and 71.2% were spontaneous (*n* = 2, 475). The trends associated with PPBMI in the incidences and risks for both medically indicated PTB and spontaneous PTB were generally consistent with those observed in overall PTB.

**Table 2.** Incidences and risks of preterm birth in different PPBMI subgroups.

	All preterm birth			Medically indicated preterm birth			Spontaneous preterm birth		
	(*n* = 3478)			(*n* = 1003)			(*n* = 2475)		
PPBMI subgroups	n (%)	aOR (95%CI)	P value	n (%)	aOR (95%CI)	P value	n (%)	aOR (95%CI)	P value
Severe underweight	41 (4.9)	1.16 (0.85-1.60)	0.35	9 (1.1)	0.90 (0.46-1.74)	0.74	32 (3.8)	1.27 (0.89-1.81)	0.20
Moderate underweight	100 (4.1)	0.96 (0.79-1.18)	0.72	28 (1.1)	0.96 (0.66-1.41)	0.83	72 (2.9)	0.97 (0.76-1.23)	0.77
Mild underweight	290 (4.7)	1.10 (0.97-1.24)	0.15	78 (1.3)	1.05 (0.83-1.33)	0.68	212 (3.4)	1.11 (0.96-1.29)	0.15
Normal weight	2291 (4.5)	Ref (1)		642 (1.2)	Ref (1)		1649 (3.2)	Ref (1)	
Overweight	594 (6.1)	1.32 (1.20-1.45)	<0.01	188 (1.9)	1.42 (1.20-1.67)	<0.01	406 (4.2)	1.27 (1.13-1.42)	<0.01
Obesity	162 (7.4)	1.47 (1.24-1.74)	<0.01	58 (2.6)	1.57 (1.17-2.09)	<0.01	104 (4.8)	1.40 (1.14-1.73)	<0.01

PPBMI: pre-pregnancy BMI, severe underweight: <16.5 kg/m^2^, moderate underweight: 16.5-17.4 kg/m^2^, mild underweight: 17.5-18.4 kg/m^2^, normal weight: 18.5-23.9 kg/m^2^, overweight: 24-27.9 kg/m^2^, obesity: PPBMI ≥ 28 kg/m^2^.

Normal weight group was used as the reference.

Analyses were adjusted for maternal age, uterine malformation, insurance status, type of conception, nulliparity, ethnicity, pregestational diabetes mellitus and chronic hypertension.

In this study, approximately 8.3% of all PTB occurred before 32 weeks of gestation (0.9% at 24-27weeks, 7.4% at 28-31weeks), while the remaining 91.7% babies born moderate to late preterm. Compared to the normal weight group, the risks of PTB in the severe, moderate and mild underweight groups didn’t differ significantly at 24-27, 28-31, and 32-36 weeks of gestation, respectively; while the risks of PTB were significantly elevated in the overweight group at 24-27 weeks of gestation (aOR, 2.52; 95% CI, 1.11-5.70), 28-31 weeks (aOR, 1.67; 95% CI, 1.23-2.28) and 32-36 weeks (aOR, 1.27; 95% CI, 1.15-1.41); the obesity group exhibited similar risks at 24-27 weeks (aOR, 3.37; 95% CI, 0.94-11.99) and 28-31 weeks (aOR, 1.70; 95% CI, 0.97-3.01); while, PTB risk was significantly higher in the obesity group at 32-36 weeks (aOR, 1.42; 95% CI, 1.19-1.70) (Table 3). However, due to the limited number of cases in the extremely/very PTB subgroups, these subgroup analyses were likely underpowered. Therefore, the corresponding estimates should be interpreted with caution and considered exploratory rather than definitive.

**Table 3.** The association between PPBMI categories and all preterm births at 24–27, 28–31, 32–36 weeks of gestation.

	PTB 24-27week			PTB 28-31week			PTB 32-36week		
	(*n* = 32)			(*n* = 257)			(*n* = 3189)		
PPBMI subgroups	n	aOR(95%CI)	P value	n	aOR(95%CI)	P value	n	aOR(95%CI)	P value
Severe underweight	0	0	0.99	2	0.89(0.22-3.59)	0.87	39	1.19(0.86-1.65)	0.30
Moderate underweight	1	1.32(0.17-9.95)	0.79	8	1.18(0.58-2.40)	0.66	91	0.95(0.76-1.17)	0.60
Mild underweight	2	1.02(0.24-4.44)	0.98	16	0.90(0.54-1.51)	0.69	272	1.11(0.98-1.27)	0.11
Normal weight	17	Ref (1)		161	Ref (1)		2113	Ref (1)	
Overweight	9	2.52(1.11-5.70)	0.03	56	1.67(1.23-2.28)	<0.01	529	1.27(1.15-1.41)	<0.01
Obesity	3	3.37(0.94-11.99)	0.06	14	1.70(0.97-3.01)	0.07	145	1.42(1.19-1.70)	<0.01

PPBMI: pre-pregnancy BMI, severe underweight: <16.5 kg/m^2^, moderate underweight: 16.5-17.4 kg/m^2^, mild underweight: 17.5-18.4 kg/m^2^, normal weight: 18.5-23.9 kg/m^2^, overweight: 24-27.9 kg/m^2^, obesity: PPBMI ≥ 28 kg/m^2^.

Normal weight group was used as the reference.

Analyses were adjusted for maternal age, uterine malformation, insurance status, type of conception, nulliparity, ethnicity, pregestational diabetes mellitus and chronic hypertension.

**Figure 2.**
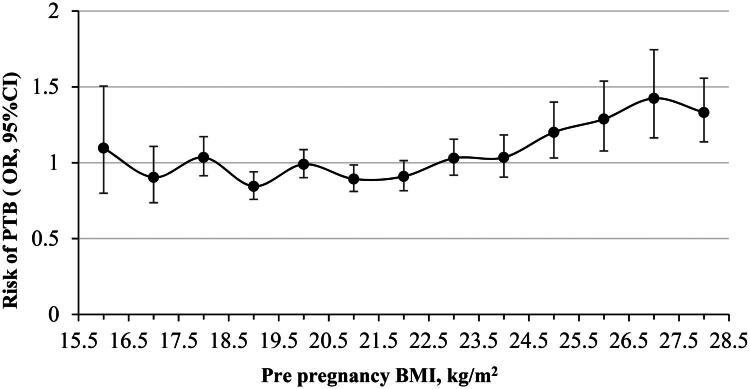
Risk of overall PTB for women in each PPBMI category vs. all other women in the study population. PPBMI was grouped into categories of 1 kg/m^2^ each, ranging from 16.5 to 27.5 kg/m^2^ or greater, PPBMI < 16.5 kg/m^2^ was grouped together. Analyses were adjusted for maternal age, uterine malformation, insurance status, type of conception, parity, ethnicity, pregestational diabetes mellitus and chronic hypertension. PPBMI: pre-pregnancy BMI; PTB: preterm birth.

Analysis using RCS demonstrated a significant nonlinear association between PPBMI and PTB risk, which followed a J-shaped curve (Figure 1). When a PPBMI of 18.5 kg/m^2^ was set as the reference, the aORs for PTB were consistent across the underweight-to-normal weight categories (Figure 1). A protective association with PTB (OR < 1) was observed for two PPBMI categories (18.5–19.5 and 20.5–21.5 kg/m^2^), both statistically significant. The association for the 19.5–20.5 kg/m^2^ category was protective but non-significant. Conversely, a significant elevation in PTB risk was observed at PPBMI values > 24.5 kg/m^2^ when each category was compared against the rest of the cohort (Figure 2).

## Discussion

### Main findings

There were three main findings in this study. First, compared to the normal weight group, underweight and severity of underweight were not significantly associated with the risk of PTB, whereas both overweight and obesity were associated with elevated risk of PTB. Second, the trends associated with PPBMI in the risks for medically indicated PTB, spontaneous PTB and PTB at different gestational weeks were generally consistent with those observed in overall PTB. Third, the association between PPBMI and PTB exhibited a J-shaped curve; The nadir of PTB risk corresponded to a PPBMI of approximately 18.5–21.5 kg/m^2^, with a potential risk threshold around 24.5 kg/m^2^.

### Strengths and limitations

This might be the largest cohort study in China investigating the association between PPBMI and PTB through a multidimensional analytical framework. We performed detailed stratification by both underweight severity and PTB subtypes (based on clinical presentation and gestational age). Furthermore, the use of RCS allowed us to robustly characterize the nonlinear relationship, delineate the PPBMI range associated with the lowest PTB risk (approximately 18.5–21.5 kg/m^2^), and define a practical threshold (around 24.5 kg/m^2^) for PTB prevention.

Despite the use of comprehensive analytical approaches and a robust sample size, our study has several limitations. First, the retrospective design relied primarily on self-reported pre-pregnancy weight data, which may cause recall bias. However, this information was collected before the occurrence of PTB, and participants were unaware of their subsequent pregnancy outcomes at the time of reporting. Therefore, any misclassification of PPBMI was likely to be non-differential with respect to PTB, which would tend to bias the associations toward the null. Despite this limitation, self-reported pre-pregnancy weight has been widely used in large-scale epidemiological studies and has shown acceptable validity. Second, the participants were predominantly recruited from urban Shanghai, and the relatively high socioeconomic and demographic homogeneity (e.g. 98.5% Han ethnicity and high insurance coverage) may limit the generalizability of our findings to women living in rural areas or those from less advantaged socioeconomic backgrounds in China. However, the distribution of PPBMI observed in this study was broadly consistent with that reported in previous studies among Chinese populations, and our findings were in line with existing evidence in Chinese women, suggesting that the results are likely representative of urban Chinese populations. Third, due to the retrospective design, we can’t systematically obtain information on factors such as active/passive smoking and alcohol consumption during early pregnancy [22,27], socioeconomic status, and history of prior preterm birth from the structured electronic medical records. Previous studies have identified these factors as potential risk factors for PTB, the missing information might cause bias in multivariate analysis.

### Interpretation

The impact of pre-pregnancy underweight on the risk of PTB remains controversial. A large US population-based study (*n* = 7,141,603) found that pre-pregnancy underweight increased the risk of PTB across different racial and ethnic populations [13]. A large retrospective cohort study (*n* = 950,356; 14.4% Asians) demonstrated an elevated risk of all PTB (including medically indicated PTB and spontaneous PTB) in underweight women, and the risk increased with the increasing severity of underweight [16]. Similarly, another cohort study (*n* = 11,726; 3.7%Asian) found a higher risk of all PTB when PPBMI was <17 kg/m^2^ [15]. However, studies predominantly enrolling Asian women have not consistently identified underweight as a significant risk factor for PTB. A large retrospective cohort study in Asian Americans [28], lacking stratification by underweight severity and PTB subgroups, found no increased risk of PTB in underweight group among Asian Americans of East Asian origin (including Chinese); In contrast, among Asian Americans of Southeast and South Asian origin, underweight was associated with a higher risk of PTB [28]. A nationwide, population-based cohort study in China, utilizing data from the NFPCP during 2010-2020 and encompassing over 8.7 million parent-child triads from both rural and urban areas, demonstrated that preconception maternal underweight was associated with an increased risk of PTB [29]; As the interval between enrollment and conception varied (1–12 months), the baseline preconception BMI may not accurately reflect maternal adiposity at conception for all participants, particularly for those conceiving ≥6 months post-enrollment, thus potentially attenuating the observed associations.

In this study, no significant association was observed between underweight severity and PTB risk. This null association was robust across PTB subtypes (medically indicated and spontaneous) and across different gestational ages, contrasting with previous results [16,30]. Meanwhile, a significantly increased PTB risk was observed among overweight/obesity women, and this association was robust across PTB subtypes (medically indicated and spontaneous). Furthermore, a consistently increased PTB risk associated with maternal overweight/obesity was observed across both extremely/very (24–31 weeks) and moderate to late (32–36 weeks) preterm birth. Thus, we concluded that the trends associated with PPBMI in the risks for spontaneous PTB, medically indicated PTB and PTB at different gestational weeks were generally consistent with those observed in overall PTB.

This study adds to the accumulating evidence that underweight was not significantly associated with the risk of PTB in East Asian populations (including Chinese) [28]. Our null finding aligns with regional studies from Southwest, Central, East, South, and North China [18,19,31–33], as well as with a meta-analysis specific to this population [34]; Conversely, the significant risk associated with overweight/obesity observed here is consistent with the broader literature. Thus, we inferred that the association between PPBMI and PTB in China exhibited a J-shaped curve, as demonstrated in this study through RCS analysis. Integrating RCS-derived nonlinear modeling with category-specific aORs revealed that PTB risk was lowest at a PPBMI of approximately 18.5–21.5 kg/m^2^, with a threshold for increased risk observed around 24.5 kg/m^2^ in this population. However, previous studies indicated that extremes of PPBMIs were significantly associated with an increased risk of PTB in American and European populations [13,15], thus, we concluded that the association between PPBMI and PTB in these populations exhibited as a U-shaped curve. The observed population-specific variations in PPBMI-PTB associations may primarily reflect racial differences, alongside disparities in environmental exposure and nutritional transitions.

Obesity and underweight are defined as conditions with excessive or not sufficient accumulation of the body fat to the extent that health is adversely affected. Obesity, especially excess visceral adiposity, is associated with elevated circulating levels of the pro-inflammatory marker C-reactive protein (CRP) [35]. During pregnancy, visceral fat mass expands, particularly in women with obesity [36], and maternal obesity further contributes to a state of chronic low-grade inflammation [37], primarily through increased secretion of adipokines from adipose tissue and heightened systemic release of pro-inflammatory cytokines [38]. It is hypothesized that this sustained pro-inflammatory environment may promote subclinical infection, thereby elevating the risk of PTB. Evidence suggests that Asian populations (including Chinese) have a higher percentage body fat for a given BMI than white populations [39,40]. This may explain why pre-pregnancy overweight alone, without progressing to obesity, was significantly associated with an increased risk of PTB in our cohort. Observational data in human populations support the theory that maternal pre-pregnancy undernutrition or lack of specific nutrients during pregnancy may influence gestation length and thus the risk of PTB [41,42]. Underweight often reflect undernutrition, chronic morbidity, and/or low socioeconomic status in previous studies. However, our population is generally healthy and well nourished. Underweight in our population may not necessarily represent undernutrition or low socioeconomic status, which may explain why underweight is not a risk factor for PTB here.

Previous studies found that risks of mortality and morbidity increase according to degree of prematurity, with babies born extremely preterm (<28 weeks of gestation) at the highest risk, followed by babies born very preterm (28-31weeks), then babies born moderate to late preterm (32-36 weeks) [3,43]. In this study, only 8.3% of preterm births occurred at less than 32 weeks of gestation, which was substantially lower than the global average level of approximately 15% (2010 to 2020) [4], however, particular attention should be given to the long-term medical and neurodevelopmental sequelae affecting born preterm neonates. Given the increasing prevalence of autism spectrum disorder (ASD) and attention-deficit hyperactivity disorder (ADHD) [44,45], alongside evidence that PTB is a significant risk factor for both ASD and ADHD [46], interventions aimed at preventing PTB could contribute to reducing the burden of these childhood mental health conditions, as no curative pharmacological treatments are currently available for either condition.

Based on these findings, clinicians may recommend maintaining a PPBMI of approximately 18.5–21.5 kg/m^2^ and provide comprehensive prenatal screening and education on PTB prevention when PPBMI exceeds 24.5 kg/m^2^.

## Conclusion

In Chinese population, pre-pregnancy underweight and severity of underweight were not significantly associated with the risk of PTB; while pre-pregnancy overweight and obesity increased the risk of PTB. The trends associated with PPBMI in the risks for medically indicated PTB, spontaneous PTB and PTB at different gestational weeks were generally consistent with those observed in overall PTB. The association between PPBMI and PTB exhibited a J-shaped curve, and the nadir of PTB risk corresponded to a PPBMI of approximately 18.5–21.5 kg/m^2^, with a potential risk threshold around 24.5 kg/m^2^.

## Supplementary Material

Supplemental Material

## Data Availability

The data that support the findings of this study are available on reasonable request from the corresponding author.
