# Supplementary material for: Association between pre-pregnancy BMI and preterm birth in Chinese women: a retrospective study
Source: Ann Med. 2026 Mar 11;58(1):2639163. doi: 10.1080/07853890.2026.2639163 (PMC12981264; doi:10.1080/07853890.2026.2639163)
Supplement: Supplemental Material [file IANN_A_2639163_SM8178.docx]

**Supplement 1. Definitions for confounding factors**

**Maternal age (years):**

According to maternal age at delivery, women in the study were classified into three groups as 18-29y, 30-34y and ≥35 y.

**Uterine malformation:**

In this study, uterine malformation is defined as a structural anatomical anomaly of the uterus resulting from abnormal development, fusion, or resorption of the Müllerian ducts during embryogenesis. The diagnosis in our cohort was primarily established through imaging assessment via transvaginal ultrasonography and/or early gynecological history, with some cases confirmed by examination of the uterine during childbirth. Women diagnosed with a septate, unicornuate, didelphic, bicornuate, or arcuate uterus, were classified as having a uterine malformation.

**Insurance status**

Women were defined as having health insurance if they were covered by any form of social medical insurance, including basic employee medical insurance, resident medical insurance, or inter-regional medical insurance.

**Type of conception**

Natural conception was defined as pregnancy achieved without the use of ovulation-inducing medications or assisted reproductive technologies.

**Parity**

Women were categorized based on parity into nulliparous and multiparous groups.

**Ethnicity**

Ethnicity was categorized as Han Chinese or ethnic minorities.

**Pregestational diabetes mellitus (PGDM)**

PGDM was diagnosed according to Guideline of diagnosis and treatment of hyperglycemia in pregnancy (2022)［Part one］(https://rs.yiigle.com/cmaid/1349426).

**Chronic hypertension**

Chronic hypertension was diagnosed according to Diagnosis and treatment of hypertension and pre-eclampsia in pregnancy: a clinical practice guideline in China (2020) (<https://rs.yiigle.com/cmaid/1192238>).

**Supplement 2. Univariate analysis between pre-pregnancy factors and PTB risk.**

|  |  | **Incidence of PTB** |  |  |
| --- | --- | --- | --- | --- |
| **Risk factor** |  | **n(%)** | **OR(95%CI)** | **P value** |
| **PPBMI** |  |  |  |  |
| normal weight | | 2291(4.5) | ref(1) |  |
| underweight |  | 431(4.5) | 0.98(0.89-1.09) | 0.75 |
| overweight |  | 594(6.1) | 1.38(1.22-1.57) | <0.01 |
| obesity |  | 162(7.4) | 1.69(1.39-2.03) | <0.01 |
| **Maternal age** |  |  |  |  |
| 18-29 y |  | 1013(4.1) | ref(1) |  |
| 30-34 y |  | 1640(4.7) | 1.17(1.08-1.27) | <0.01 |
| ≥35 y |  | 825(6.3) | 1.58(1.43-1.73) | <0.01 |
| **PGDM** |  |  |  |  |
| no |  | 3451(4.8) | ref(1) |  |
| yes |  | 27(12.4) | 2.85(1.86-4.19) | <0.01 |
| **Chronic hypertension** | |  |  |  |
| no |  | 3424(4.7) | ref(1) |  |
| yes |  | 54(15.8) | 3.78(2.79-5.02) | <0.01 |
| **Nulliparous** |  |  |  |  |
| yes |  | 2441(4.6) | ref(1) |  |
| no |  | 1037(5.4) | 1.19(1.11-1.29) | <0.01 |
| **Natural conception** | |  |  |  |
| yes |  | 3070(4.6) | ref(1) |  |
| no |  | 408(6.6) | 1.45(1.3-1.61) | <0.01 |
| **Uterine malformation** | |  |  |  |
| no |  | 3383(4.7) | ref(1) |  |
| yes |  | 95(15.3) | 3.67(2.92-4.55) | <0.01 |
| **Han ethnicity** |  |  |  |  |
| yes |  | 3416(4.8) | ref(1) |  |
| no |  | 62(5.4) | 1.15(0.88-1.47) | 0.3 |
| **Insurance** |  |  |  |  |
| yes |  | 2891(4.6) | ref(1) |  |
| no |  | 587(5.6) | 1.23(1.12-1.35) | <0.01 |

PTB: preterm birth, PPBMI: pre-pregnancy body mass index, OR:odds ratio, PGDM:pregestational diabetes mellitus.

Underweight: <18.5 kg/m^2^, normal weight: 18.5-23.9 kg/m^2^, overweight: 24.0-27.9 kg/m^2^, obesity: ≥28 kg/m^2^.
